# Effect of Two Different Dietary Weight Loss Strategies on Risk Factors for Urinary Stone Formation and Cardiometabolic Risk Profile in Overweight Women

**DOI:** 10.3390/nu14235054

**Published:** 2022-11-27

**Authors:** Roswitha Siener, Charlotte Ernsten, Norman Bitterlich, Birgit Alteheld, Christine Metzner

**Affiliations:** 1 University Stone Center, Department of Urology, University Hospital Bonn, 53127 Bonn, Germany; 2 Independent Biostatistician, Draisdorfer Str. 21, 09114 Chemnitz, Germany; 3 Department of Nutrition and Food Sciences, Nutritional Physiology, University of Bonn, 53115 Bonn, Germany; 4 Bonn Education Association for Dietetics r. A., 50935 Cologne, Germany; 5 Clinic for Gastroenterology, Metabolic Disorders and Internal Intensive Medicine (Medical Clinic III), RWTH Aachen, 52074 Aachen, Germany

**Keywords:** kidney stones, urolithiasis, calcium oxalate, uric acid, obesity, metabolic syndrome, weight reduction, diet, meal replacement, glomerular filtration rate

## Abstract

Overweight has been suggested to increase the risk of kidney stone formation. Although weight reduction might affect risk factors for urolithiasis, findings on the impact of different dietary weight loss strategies are limited. This randomized, controlled study evaluated the effect of a conventional energy-restricted modified diet with (MR group) or without meal replacement (C group) on risk factors for stone formation in overweight women without a history of urolithiasis. Of 105 participants, 78 were included into the per-protocol analysis. Anthropometric, clinical, biochemical, and 24 h urinary parameters were collected at baseline and after 12 weeks. Although both dietary interventions resulted in a significant weight reduction, relative weight loss and rate of responders were higher in the MR group. Weight loss improved cardiometabolic risk profile in both groups. Unfortunately, the benefit of decreased GPT activity in the C group was offset by a significant increase in homocysteine and a decline in GFR. While the relative supersaturation of calcium oxalate decreased significantly in both groups, a significant decline in serum uric acid concentration and relative supersaturation of uric acid was observed only in the MR group. Finally, the energy-restricted modified diet with meal replacement showed significant advantages over the energy-restricted modified diet alone.

## 1. Introduction

The prevalence of overweight and obesity has been increasing worldwide [1]. Elevated body mass index (BMI) is a major risk factor for non-communicable diseases, such as cardiovascular diseases, type 2 diabetes mellitus, musculoskeletal disorders and some cancers [1,2]. Moreover, a positive association between BMI and the risk of incident kidney stone formation has been observed in a systematic review [3]. In addition, overweight seems to increase the risk of stone recurrence [4]. A study of 163 patients followed for more than 36 months found that overweight was a strong predictor of stone recurrence in first-time stone formers [5].

Increasing BMI, which is considered a suggestive surrogate for overweight, has been reported to be related to several urinary risk factors for stone formation. Positive associations between BMI and urinary excretion of oxalate, uric acid, sodium, and phosphate and an inverse relation between BMI and urine pH were observed in both stone-forming and non-stone-forming individuals [6]. While no relation was observed between BMI and urinary supersaturation of calcium oxalate, supersaturation of uric acid increased with BMI in subjects with and without urolithiasis [6]. In contrast, a study of 527 calcium oxalate stone patients showed that the relative supersaturation of calcium oxalate increased with increasing BMI in both men and women [7].

The fundamental cause of overweight and obesity is an imbalance between energy intake and expenditure. Weight reduction is a well-established approach to overweight to improve quality of life and to reduce morbidity and mortality. However, several weight loss strategies to combat overweight and obesity may increase stone risk, including bariatric surgical procedures, such as Roux-en-Y gastric bypass and mini-gastric bypass, lipase inhibitors, and low-carbohydrate diets, such as the Atkins diet [8,9,10,11,12,13]. Therefore, a conventional energy-restricted diet may be more appropriate for weight loss, especially in patients at risk for urinary stone formation.

Obesity and type 2 diabetes mellitus are the most common causes for the development of nonalcoholic fatty liver disease (NAFLD) [14,15]. In most patients, the other components of the metabolic syndrome are also detectable [15], with a bidirectional association [16]. NAFLD, as a multi-systemic disease, favors the development of chronic kidney disease in addition to cardiovascular disease [17,18]. Several studies have also shown that patients with NAFLD have a significantly increased risk of urolithiasis [19,20].

Given the impact of overweight on the risk of urolithiasis, little is known about the effect of weight loss on 24 h urine composition and the risk of kidney stone formation. In a study of 39 obese adults with idiopathic calcium oxalate stone disease, no changes in urinary parameters were observed after short-term energy-reduced diet, except for a significant increase in urine volume and a decline in urinary supersaturation of calcium oxalate [21]. Whether weight loss affects urinary risk factors for kidney stone formation in the non-stone-forming population remains unclear. Moreover, studies on the effect of a conventional energy-restricted diet incorporating meal replacement on 24 h urine composition and the stone risk are lacking. Therefore, the aim of the present study was to evaluate the effect of two different weight loss strategies on risk factors for urinary stone formation and cardiometabolic risk profile in overweight women without a history of urolithiasis.

## 2. Materials and Methods

### 2.1. Participants

Women aged 18 to 60 years with a BMI between 27.0 and 39.9 kg/m^2^ were recruited from two outpatient nutrition education centers in Berlin and Frankfurt/M, Germany. Study participants were enrolled between October 2004 and March 2006. Only women were included to ensure a homogeneous group. The inclusion criterion was the presence of at least one of the following blood lipid abnormalities: serum triglycerides (TG) 150–400 mg/dL, total cholesterol (TC) ≥ 200 mg/dL, HDL-cholesterol (HDL-C) ≤ 50 mg/dL, LDL-cholesterol (LDL-C) ≥ 175 mg/dL. Exclusion criteria were pharmacological treatment of diabetes, anticoagulant therapy, intake of mineral or vitamin supplements, hyper- or hypothyroidism, cardiac pacemaker, protein or lactose intolerance, and contraindications to exercise. The study was approved by the Ethics Committees of the Chamber of Physicians of the German Federal States of Hessen and Berlin, and informed consent was obtained from each participant prior to the start of the study. The study was registered with ClinicalTrials.gov (NCT03109834).

### 2.2. Study Design

This open-label, randomized and controlled intervention trial randomly assigned subjects to the control (C) or the meal replacement (MR) group. The study was conducted with the aim of investigating the effect of a conventional energy-restricted modified diet with or without meal replacement on weight loss and cardiometabolic risk profile in overweight women [22]. Data from 24 h urine collections and blood samples were evaluated at baseline and after 12 weeks of dietary weight loss intervention for changes in risk factors for urinary stone formation.

Both groups were instructed to follow an energy-restricted lacto-vegetarian-oriented diet with a balanced selection of nutrient-dense foods at approximately 1200 kcal per day. The C group was recommended a conventional energy-restricted modified diet containing 15–20% of energy intake in the form of protein, 30% of energy intake in the form of fat, and 50–55% of energy intake in the form of carbohydrates. The MR group was advised to replace two of three meals per day with meal replacement products [22].

All participants attended 10 nutrition education-training sessions in groups of 8 to 10 women. Training sessions were conducted separately for each group by a nutritionist and lasted one hour each. Topics included practical knowledge of meal frequency, portion sizes, and food selection with the goal of changing lifestyle and dietary behavior. All participants received instruction manuals with recipes, a sample meal plan, and physical activity information.

### 2.3. Anthropometric and Clinical Measurements

Anthropometric measurements and venous blood sampling were performed in the morning after at least 12 h of overnight fasting. Body mass index (BMI, kg/m^2^) was calculated by dividing body weight (kg) by the square of height (m).

Waist, hip, and thigh circumference were determined using a flexible tape measure. Body fat mass (BFM), body cell mass (BCM) and lean body mass (LBM) were determined under standardized conditions by bioelectrical impedance analysis (BIA; AKERN BIA device, Italy) using the biaform^®^ software version 2.2. Blood pressure was measured under standardized conditions after a 10 min rest period. Blood samples were collected at 9:00 a.m. for analysis of serum cortisol. Twenty-four hour urine samples were collected at baseline and after the 12-week dietary intervention.

### 2.4. Laboratory Methods

Serum uric acid (uricase-PAP method), glucose (hexokinase method), TG (GPO-PAP method; Olympus 600 analyzer), TC (CHOD-PAP method), LDL-C and HDL-C (enzymatic color test), gamma-glutamyltransferase (kinetic color test IFCC), folic acid, homocysteine, and cortisol (chemiluminiscence method; ADVIAR Centaur analyzer), and glycated hemoglobin (HbA1c) (HPLC; Biorad Variant II analyzer) were analyzed by the medical laboratory Potsdam, Germany.

The total antioxidative capacity (TAC) was determined by the reaction of antioxidants in the sample with a defined amount of exogenously provided hydrogen peroxide (H_2_O_2_). The antioxidants in the sample eliminate a certain amount of the provided H_2_O_2_. The residual H_2_O_2_ is determined photometrically by an enzymatic reaction, which involves the conversion of TMB to a colored product.

Urine volume, density, pH (potentiometry) and the concentrations of sodium, chloride, potassium (ion-selective electrode), calcium (cresolphthalein complex), magnesium (methylthymol blue), inorganic sulfate (nephelometry), inorganic phosphate (phosphate molybdate reaction), ammonium (ion selective electrode), creatinine (Jaffé reaction), citrate (enzymatically, citrate lyase), uric acid (enzymatically, uricase) and oxalate (enzymatically, oxalate oxidase) were determined at the laboratory of the University Stone Center, Department of Urology, University Hospital Bonn, Germany. The risk of stone formation, computed as relative supersaturation of calcium oxalate, uric acid, brushite, and struvite, was calculated using the computer program EQUIL2 (University of Florida, Gainesville, FL, USA) [23]. Laboratory quality certification was available for each parameter, except for folic acid, homocysteine, and TAC.

### 2.5. Statistical Methods

Statistical comparisons between groups were performed with the nonparametric Mann–Whitney U test for unpaired data. The nonparametric Wilcoxon test was used to analyze changes before and after intervention. Parametric *t*-tests were performed as sensitivity analysis to confirm the results. Correlations between variables were calculated using Spearman’s rank correlation. Comparisons between categorical variables were assessed using Fisher’s exact test. Contingency tables and comparisons between categorical variables with more than two categories were evaluated with the Fisher-Freeman-Halton test. All statistical tests were two-tailed for the exclusively explorative analysis. Differences were considered significant at *p* < 0.05, without taking into account the effects of multiple testing. Data are presented as mean ± standard deviation. Statistical analysis was performed using SPSS^®^ for Windows, version 27.0 (IBM, Armonk, New York, NY, USA).

## 3. Results

### 3.1. Participants

**Figure 1:**
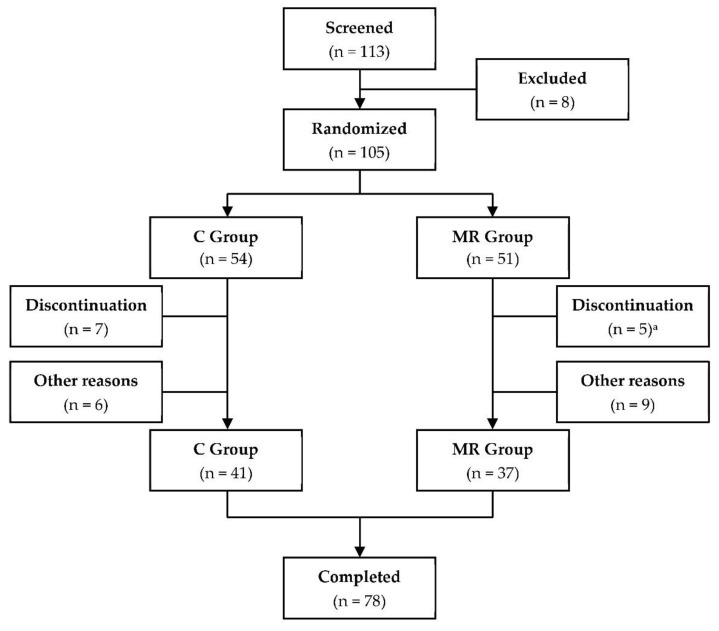
Trial profile. Abbreviations: C, control; MR, meal replacement; ^a^ one adverse event.

A total of 105 overweight women were randomized, of whom 78 were included into the per protocol analysis (Figure 1). Twelve women discontinued the study, eleven for personal reasons (seven women in the C group and four women in the MR group) and one due to adverse effects (diarrhea). Additionally, 15 women had to be excluded from analysis due to lack of 24 h urine samples. Meal replacements were well tolerated by the subjects.

Baseline characteristics did not differ between both groups (Table 1). Approximately 75% of women in both groups had obesity stage I (30.0–34.9 kg/m^2^) or stage II (35.0–39.9 kg/m^2^).

### 3.2. Anthropometric and Clinical Characteristics

The dietary intervention resulted in significant absolute weight loss in the C group and the MR group at 12 weeks, with no significant difference between groups (Table 2). In contrast, relative weight loss and rate of responders (weight loss > 5%) were significantly higher in the MR group compared with the C group at 12 weeks. In addition, fatty liver index (FLI), BMI, waist, hip and thigh circumference decreased significantly and to a similar extent in both groups during the dietary intervention. While BFM (kg and %), BCM (kg), and LBM (kg) were significantly lower, BCM (%), LBM (%), and ECM (%) were significantly higher in the C group and MR group after 12 weeks of dietary intervention. Systolic and diastolic blood pressure declined significantly in both groups.

### 3.3. Cardiometabolic Risk Profile

The changes in the cardiometabolic risk profile are presented in Table 3. The number of women with a waist circumference > 88 cm and the number of participants with a FLI ≥ 60 decreased significantly in both groups, while the frequency of systolic blood pressure ≥ 130 mmHg, diastolic blood pressure ≥ 85 mmHg or blood pressure ≥ 130/85 mmHg declined significantly only in the MR group.

### 3.4. Clinical Chemistry and Biochemical Characteristics

Dietary weight loss intervention resulted in a significant decline in TC, LDL-C, but also in HDL-C in the C group and the MR group (Table 4). No changes were observed in serum glucose, HbA1c and TG between and within the two groups after 12 weeks. Serum cortisol concentration increased significantly and TAC remained unchanged in the C group and the MR group. While GGT activity decreased significantly in both groups, GPT activity declined significantly only in the C group. Serum uric acid was reduced and folic acid increased only in the MR group at 12 weeks. Homocysteine and creatinine increased, while GFR declined during dietary weight loss intervention only in the C group.

After weight loss intervention, serum uric acid was correlated with GPT (C group: R = 0.401, *p* = 0.028; MR group: R = 0.384, *p* = 0.043), GGT (C group: R = 0.427, *p* = 0.018; MR group: R = 0.569, *p* = 0.002), and FLI (C group: R = 0.430, *p* = 0.020; MR group: 0.628, *p* < 0.001) in both groups. A significant positive correlation between serum uric acid and GOT (R = 0.495, *p* = 0.005), HbA1c (R = 0.409, *p* = 0.025), and serum creatinine (R = 0.396, *p* = 0.030), and a negative correlation with serum cortisol (R = −0.375, *p* = 0.041) at 12 weeks was observed only in the C group. Serum uric acid was inversely correlated with HDL-C (R = −0.441, *p* = 0.019), and urine volume (R = −0.470, *p* = 0.012) and positively associated with TG (R = 0.603, *p* = 0.001), and TAC (R = 0.519, *p* = 0.005) after dietary weight loss intervention only in the MR group.

### 3.5. Urine Composition

Urine volume increased significantly in the C group and the MR group during the 12 weeks of weight loss intervention (Table 5). The relative supersaturation of calcium oxalate decreased significantly during intervention in both groups, without inter-group differences. Urine density and the relative supersaturation of uric acid declined significantly only in the MR group. No change in any other urinary parameter was observed.

No correlation was observed between BMI and 24 h urinary parameters, except for a significantly inverse association between BMI and urinary citrate excretion in the C group after 12 weeks (baseline: R = 0.027, *p* = 0.866; 12 weeks: R = −0.381, *p* = 0.014). Moreover, no correlation was found between BMI and GFR, either before (C baseline: R = 0.192, *p* = 0.311; MR baseline: R = 0.036, *p* = 0.857) or after dietary intervention (C 12 weeks: R = 0.174, *p* = 0.357; MR 12 weeks: 0.280, *p* = 0.149).

## 4. Discussion

The present study provides the first findings on the effect of two different weight loss strategies on metabolic risk factors for kidney stone formation in individuals without urolithiasis. Both dietary interventions, the conventional energy-restricted diet alone or with meal replacement, had a significant beneficial effect on weight loss of women. As indicated by the significantly higher relative weight loss and rate of responders, weight loss was even more pronounced in the MR group. Evaluation of 24 h urine composition revealed a significantly increased urine volume in both groups. Both dietary weight loss strategies were able to decrease the relative supersaturation of calcium oxalate to a significant and similar extent. It is suggested that the significant reduction in the relative supersaturation of calcium oxalate is due to the significant increase in urine volume in both groups. The results of the present study are in accordance with previous findings in kidney stone formers. A study of 39 obese calcium oxalate stone patients found a significant increase in urinary volume and a significant decline in the supersaturation of calcium oxalate, calculated as Tiselius ion-activity product (AP) index, after a low-calorie diet for 12 weeks [21]. Moreover, in the present study, a significant decline in urine density and a significant reduction in the relative supersaturation of uric acid were observed only in the MR group. In addition to the increase in urine volume, the decrease in the relative supersaturation of uric acid can probably be attributed to the decline in urine density and the mild but non-significant increase in urinary pH in the MR group.

Prior data suggested that increasing BMI is associated with the urinary excretion of several lithogenic factors in non-stone-forming individuals, including increased urinary oxalate, uric acid, sodium, and phosphate, and decreased urinary pH [6]. In the present study, no relationship was observed between BMI and 24 h urinary parameters, except for a significantly negative correlation with urinary citrate excretion after dietary intervention in the C group.

Interestingly, serum uric acid concentration decreased significantly after weight reduction only in the MR group. Besides dietary risk factors such as consumption of animal-derived purine-rich foods, legumes, and alcoholic beverages [31,32,33], serum uric acid concentration is known to be associated with metabolic syndrome [34,35]. Many studies have shown that serum uric acid is also associated with the risk for developing NAFLD [36,37,38,39]. A longitudinal cohort study reported that serum uric acid levels were positively correlated with higher serum ALT (GPT), AST (GOT), and TG, and inversely associated with serum HDL-C levels in men with NAFLD [40]. Moreover, the data supported a role in lower serum uric acid concentrations leading to improvement in fatty liver disease [40]. In the present study, serum uric acid was positively correlated with FLI in both groups after weight loss intervention. In addition, serum uric acid was positively associated with GPT, GGT, and TG, and inversely correlated with HDL-C in the MR group after weight loss intervention. These findings are consistent with the observation from a study of 31 hyperuricemic patients with NAFLD, which found that treatment with allopurinol improved concentrations of serum GPT, GOT, TC, and TG [41].

Metabolic syndrome and its components, including abdominal obesity, hypertension, diabetes mellitus, and dyslipidemia, are associated with elevated risk of cardiovascular disease and may increase the risk of stone formation [42,43,44,45]. A study of 148 individuals without a history of kidney stone disease revealed that urine pH values decreased with increasing number of metabolic syndrome traits [46]. In stone formers, a negative correlation between BMI and urinary pH has been reported in both men and women [7]. Moreover, a recent study of stone formers indicated that all components of the metabolic syndrome except hyperlipidemia were independently associated with low urinary pH suggesting a mechanism independent from insulin resistance [47]. In the present study, the prevalence of metabolic syndrome did not differ between the two groups at either time point.

Because a history of urolithiasis is potentially related to an increased risk of cardiovascular disease [48,49], the effect of the two different weight loss strategies on cardiometabolic risk factors was also examined. In the present study, weight loss resulted in a significant decline in systolic and diastolic blood pressure in both groups. A significant and similar decrease in TC and LDL-C, but also in HDL-C was observed in both intervention groups. After dietary weight loss intervention, GPT activity was significantly lower only in the C group. However, a significant higher serum homocysteine was observed in the C group compared with the MR group after intervention. Overall, cardiometabolic risk profile was more favorably affected by the energy-restricted modified diet with meal replacement than by the energy-restricted modified diet alone.

In this study, the conventional energy-restricted modified diet was found to be associated with a significant increase in serum creatinine and a concomitant decrease in GFR. The effect of weight loss diets on GFR is of major clinical interest, particularly with regard to experimental findings suggesting that high-protein diets may have adverse effects on kidney health [50,51]. In addition, increased BMI may also be associated with an elevation in GFR, a phenomenon termed glomerular hyperfiltration [52]. In the present study, urinary sulfate, phosphate and uric acid excretion, markers of protein intake, remained unchanged after the dietary intervention in both groups, suggesting that GFR was not affected by protein intake. Because no correlation was found between BMI and GFR, it is assumed that the decrease in GFR in the C group was not associated with a reduction in glomerular hyperfiltration due to weight loss. Further research is needed to evaluate longer-term effects of dietary weight loss strategies on renal function and the impact of a conventional energy-restricted modified diet with or without meal replacement on the cardiometabolic risk profile and urinary risk factors for stone formation in patients with a history of urolithiasis.

The study has potential limitations. Although urolithiasis is more prevalent in men, only overweight women were included in the study. Only non-stone formers were recruited and, presumably, the parameters differ between stone formers and non-stone formers. Furthermore, clinical parameters were evaluated only once after three months, so the urine composition may not be representative. Another limitation of the study includes the lack of a longer follow-up. Finally, diets other than the balanced lacto-vegetarian-oriented diet studied, such as the DASH diet or the Mediterranean diet, might have been an option to improve urinary parameters. However, randomized, controlled trials on the effects of dietary weight loss strategies on cardiometabolic risk profile and 24 h urinary parameters in overweight adults with or without a history of urolithiasis are lacking. Therefore, we believe that our randomized, controlled study provides an important addition to the existing literature.

## 5. Conclusions

The results of this study show that both dietary weight loss strategies are safe and effective approaches for weight reduction. Compliance was higher in the MR group than in the C group, as evidenced by the higher relative weight loss and rate of responders. Both dietary interventions were able to decrease the relative supersaturation of calcium oxalate to a significant and similar extent. In addition, the energy-restricted modified diet with meal replacement resulted in a significant decline in serum uric acid concentration and the relative supersaturation of uric acid. Cardiometabolic risk profile was favorably influenced in both groups, mainly by the decrease in blood pressure, TC, LDL-C, and GGT activity. Unfortunately, the benefit of decreased GPT activity in the C group was offset by a significant increase in homocysteine and a significant decline in GFR. Finally, the energy-restricted modified diet with meal replacement showed significant advantages over the energy-restricted modified diet alone.

## Figures and Tables

**Table 1:** Baseline characteristics of overweight women.

	C Group n = 41 Mean ± SD/n (%)	MR Group n = 37 Mean ± SD/n (%)	C vs. MR *p* Value
Age (years)	50.2 ± 11.0	49.0 ± 12.8	0.821 ^a^
Weight (kg)	86.2 ± 9.6	85.8 ± 8.9	0.790 ^a^
Height (cm)	164.4 ± 6.2	164.3 ± 6.0	0.982 ^a^
BMI (kg/m^2^)	31.8 ± 2.2	31.7 ± 2.2	0.811 ^a^
BMI 27.0–29.9 kg/m^2^ (n)	9 (22.0%)	9 (24.3%)	0.867 ^b^
BMI 30.0–34.9 kg/m^2^ (n)	28 (68.3%)	26 (70.3%)	0.867 ^b^
BMI 35.0–39.9 kg/m^2^ (n)	4 (9.8%)	2 (5.4%)	0.867 ^b^
WC (cm)	96.4 ± 6.9	95.5 ± 7.4	0.681 ^a^
WC 80–88 cm (n)	6 (14.6%)	7 (18.9%) ^c^	0.762 ^d^
WC > 88 cm (n)	35 (85.4%)	29 (78.4%) ^c^	0.762 ^d^
BP systolic (mmHg)	130.0 ± 19.4	132.3 ± 18.2	0.262 ^a^
BP diastolic (mmHg)	80.6 ± 9.2	80.4 ± 10.2	0.714 ^a^
Glucose (mg/dL) ^e^	87.9 ± 11.1	90.9 ± 14.2	0.494 ^a^
TC (mg/dL) ^e^	238.1 ± 35.2	235.9 ± 51.1	0.893 ^a^
HDL-C (mg/dL) ^e^	58.7 ± 11.9	56.9 ± 15.6	0.401 ^a^
TG (mg/dL) ^f^	155.0 ± 86.5	207.6 ± 158.2	0.425 ^a^
Serum uric acid (mg/dL) ^e^	4.92 ± 0.99	5.17 ± 1.28	0.451 ^a^
Smokers (n)	7 (17.1%)	6 (16.2%)	1.000 ^d^

Abbreviations: BMI, body mass index; BP, blood pressure; C, control; HDL-C, HDL cholesterol; MR, meal replacement; SD, standard deviation; TC, total cholesterol; TG, triglycerides; WC, waist circumference. ^a^ *p*-value: Mann–Whitney-U-test. ^b^ *p*-value: Fisher-Freeman-Halton test. ^c^ n = 36; one patient with a WC of 79 cm. ^d^ *p*-value: Fisher’s exact test. ^e^ biochemical measurements with the same method: C (n = 30), MR (n = 28). ^f^ biochemical measurements with the same method, data not available for all women: C (n = 29), MR (n = 28).

**Table 2:** Anthropometric and clinical characteristics in overweight women at baseline and after dietary intervention.

		C Group n = 41 Mean ± SD n (%)			MR Group n = 37 Mean ± SD n (%)		C vs. MR
	Baseline	Week 12	*p* Value ^a^	Baseline	Week 12	*p* Value ^a^	*p* Value ^b^
Weight (kg)	86.2 ± 9.6	81.2 ± 8.9	<0.001	85.8 ± 8.9	79.4 ± 8.9	<0.001	0.072
Relative weight (%)	100 ± 0	94.2 ± 4.1	<0.001	100 ± 0	92.5 ± 3.1	<0.001	0.048
Weight loss > 5% (n/%)	–	22 (53.7%)	–	–	29 (78.4%)	–	0.032 ^c^
BMI (kg/m^2^)	31.8 ± 2.2	30.0 ± 2.2	<0.001	31.7 ± 2.2	29.4 ± 2.4	<0.001	0.068
Waist circumference (cm)	96.4 ± 6.9	91.0 ± 7.0	<0.001	95.5 ± 7.4	88.5 ± 7.7	<0.001	0.086
Hip circumference (cm) ^d^	115.0 ± 6.4	110.1 ± 6.8	<0.001	114.3 ± 6.3	108.7 ± 5.9	<0.001	0.623
Thigh circumference (cm)	64.6 ± 4.8	62.0 ± 5.1	<0.001	64.3 ± 5.0	61.0 ± 5.0	<0.001	0.124
WHtR	0.587 ± 0.043	0.554 ± 0.047	<0.001	0.581 ± 0.044	0.539 ± 0.048	<0.001	0.082
WHR ^d^	0.84 ± 0.05	0.84 ± 0.06	0.428	0.83 ± 0.05	0.82 ± 0.06	0.044	0.344
VAI ^e,f^	2.28 ± 1.44	2.22 ± 1.16	0.798	3.49 ± 3.26	2.96 ± 2.48	0.316	0.596
FLI ^e,g^	65.6 ± 15.9	53.1 ± 18.8	<0.001	69.2 ± 21.8	52.4 ± 24.4	<0.001	0.212
BFM (kg)	38.2 ± 6.7	34.0 ± 6.3	<0.001	37.7 ± 6.4	32.7 ± 6.5	<0.001	0.110
BFM (%)	44.0 ± 3.7	41.7 ± 4.1	<0.001	43.7 ± 3.6	40.9 ± 4.3	<0.001	0.209
BCM (kg)	25.8 ± 2.6	25.0 ± 2.5	<0.001	25.7 ± 2.5	24.7 ± 2.4	<0.001	0.681
BCM (%)	30.1 ± 3.0	30.9 ± 3.0	<0.001	30.1 ± 2.7	31.3 ± 3.3	<0.001	0.216
LBM (kg)	48.3 ± 4.5	47.5 ± 3.9	<0.001	48.4 ± 3.6	47.0 ± 3.5	<0.001	0.131
LBM (%)	56.3 ± 3.8	58.7 ± 4.2	<0.001	56.6 ± 3.5	59.5 ± 4.3	<0.001	0.324
ECM (kg)	22.6 ± 2.7	22.5 ± 2.3	0.942	22.7 ± 2.2	22.3 ± 2.3	0.085	0.222
ECM (%)	26.2 ± 1.9	27.8 ± 2.2	<0.001	26.5 ± 2.3	28.2 ± 2.6	<0.001	0.811
BP systolic (mmHg)	130.0 ± 19.4	120.1 ± 13.9	0.014	132.3 ± 18.2	119.6 ± 13.8	0.001	0.183
BP diastolic (mmHg)	80.6 ± 9.2	75.5 ± 8.3	0.009	80.4 ± 10.2	76.0 ± 8.6	0.036	0.752
Heart rate (1/min) ^h^	78.1 ± 11.6	76.0 ± 12.6	0.145	79.1 ± 12.8	76.9 ± 10.5	0.292	0.986

Abbreviations: BCM, body cell mass; BFM, body fat mass; BMI, body mass index; BP, blood pressure; C, control; ECM, extracellular mass; FLI, fatty liver index; LBM, lean body mass; MR, meal replacement; SD, standard deviation; VAI, visceral adiposity index; WHR, Waist-to-hip ratio; WHtR, Waist-to-height ratio. ^a^ *p*-value: Wilcoxon-test within groups. ^b^ *p*-value: Mann–Whitney-U-test. ^c^ *p*-value: Fisher’s exact test. ^d^ data not available for all women: C (n = 30), MR (n = 27). ^e^ biochemical measurements with the same method, data not available for all women: C (n = 29), MR (n = 28). ^f^ VAI calculated according to [24]. ^g^ FLI calculated according to [25]. ^h^ data not available for all women: C (n = 41), MR (n = 36).

**Table 3:** Changes in the cardiometabolic risk profile in overweight women at baseline and after dietary intervention.

		C Group n = 41 n (%)			MR Group n = 37 n (%)		C vs. MR
	Baseline	Week 12	*p* Value ^a^	Baseline	Week 12	*p* Value ^a^	*p* Value ^b^
Weight reduction > 5%	–	22 (53.7%)	–	–	29 (78.4%)	–	0.032
WC > 88 cm	35 (85.4%)	26 (63.4%)	0.041	29 (78.4%)	19 (51.4%)	0.027	0.622
BP systolic ≥ 130 mmHg	23 (56.1%)	16 (39.0%)	0.184	25 (67.6%)	12 (32.4%)	0.005	0.699
BP diastolic ≥ 85 mmHg	12 (29.3%)	7 (17.1%)	0.295	14 (37.8%)	5 (13.5%)	0.032	0.858
BP ≥ 130/85 mmHg	11 (26.8%)	7 (17.1%)	0.424	13 (35.1%)	4 (10.8%)	0.025	0.748
TG ≥ 150 mg/dL ^c^	11 (37.9%)	13 (44.8%)	0.790	14 (50.0%)	15 (53.6%)	1.000	0.747
HDL-C < 50 mg/dL ^d^	9 (30.0%)	10 (33.3%)	1.000	10 (35.7%)	11 (39.3%)	1.000	0.921
Fasting glucose ≥ 110 mg/dL ^d^	2 (6.7%)	0 (0%)	0.492	2 (7.1%)	1 (3.6%)	1.000	0.942
FLI ≥ 60 ^c,e^	20 (69.0%)	10 (34.5%)	0.017	20 (71.4%)	11 (39.3%)	0.031	0.980
Hypertriglyceridemic waist ^c,f^	10 (34.5%)	8 (27.6%)	0.777	12 (42.9%)	8 (28.6%)	0.403	0.947
Atherogenic dyslipidemia ^c,g^	0 (0%)	0 (0%)	–	2 (7.1%)	3 (10.7%)	1.000	0.159
Metabolic syndrome ^c,h^	13 (44.8%)	9 (31.0%)	0.417	17 (60.7%)	10 (35.7%)	0.108	0.669
Metabolic syndrome ^c,i^	5 (17.2%)	3 (10.3%)	0.706	8 (28.6%)	6 (21.4%)	0.758	0.536
Metabolic syndrome ^c,j^	14 (48.3%)	13 (44.8%)	1.000	17 (60.7%)	14 (50.0%)	0.591	0.800

Abbreviations: BP, blood pressure; C, control; FLI, fatty liver index; HDL-C, HDL-cholesterol; MR, meal replacement; TG, triglycerides; WC, waist circumference. ^a^ *p*-value: Fisher’s exact test within groups. ^b^ *p*-value: Fisher-Freeman-Halton test. ^c^ biochemical measurements with the same method, data not available for all women: C (n = 29), MR (n = 28). ^d^ biochemical measurements with the same method: C (n = 30), MR (n = 28). ^e^ FLI calculated according to [25]. ^f^ hypertriglyceridemic waist, i.e., WC > 88 cm and TG > 150 mg/dL [26]. ^g^ atherogenic dyslipidemia, i.e., HDL-C < 40 mg/dL and TG ≥ 150 mg/dL [27]. ^h^ defined by AHA/NHLBI criteria for metabolic syndrome [28]. ^i^ defined by NCEP-ATP III criteria for metabolic syndrome [27]. ^j^ defined by IDF criteria for metabolic syndrome [29].

**Table 4:** Clinical chemistry and biochemical characteristics in overweight women at baseline and after dietary intervention.

		C Group n = 30 ^a^ Mean ± SD			MR Group n = 28 ^a^ Mean ± SD		C vs. MR
	Baseline	Week 12	*p* Value ^b^	Baseline	Week 12	*p* Value ^b^	*p* Value ^c^
Total protein (g/dL)	7.45 ± 0.35	7.48 ± 0.47	0.843	7.37 ± 0.35	7.54 ± 0.54	0.238	0.549
Glucose (mg/dL)	87.9 ± 11.1	86.3 ± 10.4	0.506	90.9 ± 14.2	90.4 ± 10.8	0.625	0.760
HbA1c (%)	5.59 ± 0.48	5.55 ± 0.40	0.657	5.59 ± 0.43	5.59 ± 0.46	0.986	0.777
TC (mg/dL)	238.1 ± 35.2	226.3 ± 42.6	0.015	235.9 ± 51.1	221.1 ± 53.0	0.028	0.960
HDL-C (mg/dL)	58.7 ± 11.9	55.5 ± 11.5	0.005	56.9 ± 15.6	54.6 ± 14.2	0.005	0.856
LDL-C (mg/dL) ^d^	159.2 ± 24.9	148.4 ± 31.8	0.009	154.0 ± 38.6	140.9 ± 38.5	0.001	0.940
TG (mg/dL) ^d^	155.0 ± 86.5	150.0 ± 65.1	0.643	207.6 ± 158.2	178.6 ± 121.7	0.287	0.773
TG/HDL-C ^d^	2.79 ± 1.72	2.78 ± 1.37	0.949	4.23 ± 4.03	3.69 ± 3.03	0.508	0.596
LDL-C/HDL-C ^d^	2.80 ± 0.63	2.73 ± 0.67	0.442	2.85 ± 0.85	2.69 ± 0.80	0.026	0.345
TC/HDL-C	4.2 ± 0.8	4.2 ± 0.8	0.839	4.4 ± 1.2	4.2 ± 1.1	0.327	0.382
GOT (U/L)	24.7 ± 6.5	23.1 ± 5.7	0.132	24.4 ± 6.4	24.4 ± 6.4	0.557	0.596
GPT (U/L)	29.1 ± 15.6	25.3 ± 13.9	0.045	29.4 ± 13.5	26.7 ± 11.7	0.302	0.719
GGT (U/L)	28.4 ± 13.6	24.8 ± 11.4	<0.001	34.9 ± 29.2	29.0 ± 20.5	0.002	0.455
Uric acid (mg/dL)	4.92 ± 0.99	4.78 ± 0.98	0.407	5.17 ± 1.28	4.87 ± 1.21	0.013	0.312
Homocysteine (µmol/L)	10.5 ± 2.0	11.7 ± 2.7	0.035	12.6 ± 4.9	11.8 ± 3.6	0.155	0.010
Folic acid (µg/L)	11.4 ± 4.5	12.3 ± 7.7	0.931	10.0 ± 4.5	14.6 ± 6.1	<0.001	0.005
Creatinine (mg/dL)	0.87 ± 0.08	0.91 ± 0.07	0.001	0.89 ± 0.12	0.90 ± 0.11	0.486	0.071
GFR (mL/min/1.73 m^2^) ^e^	78.6 ± 11.6	74.6 ± 10.3	0.001	79.6 ± 15.0	79.0 ± 14.9	0.452	0.067
CRP (mg/L) ^f^	4.4 ± 3.5	5.0 ± 5.6	0.726	5.2 ± 3.8	5.3 ± 4.4	0.883	0.883
TSH (mU/L) ^g^	1.39 ± 0.91	1.40 ± 0.76	0.871	1.52 ± 0.91	1.63 ± 0.96	0.146	0.368
TAC (µmol/L)	282.8 ± 42.3	294.2 ± 38.0	0.455	270.0 ± 26.5	284.0 ± 33.7	0.085	0.570
Cortisol (nmol/L)	366.9 ± 143.3	480.9 ± 144.2	<0.001	431.4 ± 150.9	562.9 ± 171.1	<0.001	0.685
TAC/Cortisol (10^3^)	0.91 ± 0.45	0.68 ± 0.26	0.006	0.72 ± 0.29	0.56 ± 0.20	0.001	0.740

Abbreviations: C, control; CRP, C-reactive protein; GFR, glomerular filtration rate; GGT, gamma-glutamyltransferase; GOT, glutamic oxaloacetic transaminase; GPT, glutamic-pyruvic transaminase; HbA1c, glycated hemoglobin; HDL-C, HDL cholesterol; LDL-C, LDL cholesterol; MR, meal replacement; SD, standard deviation; TAC, total antioxidative capacity; TC, total cholesterol; TG, triglycerides; TSH, thyroid-stimulating hormone. ^a^ biochemical measurements with the same method. ^b^ *p*-value: Wilcoxon-test within groups. ^c^ *p*-value: Mann–Whitney-U-test. ^d^ data not available for all women: C (n = 29), MR (n = 28). ^e^ calculated according to the CKD-EPI equation [30]. ^f^ data not available for all women: C (n = 30), MR (n = 27). ^g^ data not available for all women: C (n = 29), MR (n = 26).

**Table 5:** Twenty-four-hour urine composition in overweight women at baseline and after 12-weeks weight loss intervention.

		C Group n = 41 Mean ± SD			MR Group n = 37 Mean ± SD		C vs. MR
	Baseline	Week 12	*p* Value ^a^	Baseline	Week 12	*p* Value ^a^	*p* Value ^b^
Volume (L/24 h)	2.113 ± 0.753	2.404 ± 0.801	0.018	2.114 ± 0.669	2.764 ± 1.143	< 0.001	0.211
pH	6.38 ± 0.62	6.31 ± 0.63	0.890	6.19 ± 0.52	6.41 ± 0.57	0.062	0.232
Density (g/cm^3^)	1.007 ± 0.004	1.006 ± 0.004	0.269	1.008 ± 0.004	1.006 ± 0.003	0.001	0.184
Sodium (mmol/24 h)	163 ± 82	153 ± 67	0.756	165 ± 71	146 ± 85	0.083	0.299
Potassium (mmol/24 h)	57 ± 27	62 ± 31	0.387	62 ± 22	67 ± 35	0.907	0.617
Calcium (mmol/24 h)	3.53 ± 1.84	3.74 ± 2.31	0.555	4.17 ± 2.30	4.64 ± 2.84	0.941	0.850
Magnesium (mmol/24 h)	3.25 ± 1.80	3.10 ± 1.73	0.553	3.56 ± 1.64	3.67 ± 2.12	0.665	0.931
Ammonium (mmol/24 h)	20.1 ± 9.8	21.1 ± 10.1	0.368	21.0 ± 11.0	20.9 ± 11.5	0.743	0.324
Chloride (mmol/24 h)	178 ± 90	177 ± 71	0.729	185 ± 77	172 ± 96	0.172	0.198
Phosphate (mmol/24 h)	24.0 ± 10.2	23.9 ± 11.2	0.848	24.9 ± 9.8	24.1 ± 14.7	0.094	0.269
Sulfate (mmol/24 h)	18.0 ± 7.7	18.1 ± 8.2	0.837	20.5 ± 8.5	22.6 ± 9.6	0.464	0.577
Creatinine (mmol/24 h)	9.04 ± 4.12	8.70 ± 3.95	0.980	9.69 ± 3.50	8.83 ± 5.59	0.070	0.212
Uric acid (mmol/24 h)	2.78 ± 1.21	2.97 ± 1.10	0.322	3.02 ± 1.27	2.85 ± 1.01	0.261	0.148
Oxalate (mmol/24 h)	0.349 ± 0.180	0.289 ± 0.126	0.226	0.333 ± 0.106	0.297 ± 0.155	0.068	0.584
Citrate (mmol/24 h)	3.100 ± 1.792	3.050 ± 1.757	0.848	2.891 ± 1.672	3.014 ± 1.983	0.988	1.000
RS Brushite	0.575 ± 0.353	0.550 ± 0.630	0.148	0.630 ± 0.506	0.626 ± 0.461	0.929	0.319
RS Struvite	0.066 ± 0.130	0.064 ± 0.119	0.788	0.035 ± 0.051	0.040 ± 0.049	0.602	0.605
RS Uric acid	0.714 ± 0.672	0.851 ± 1.289	0.599	1.021 ± 0.983	0.547 ± 0.596	0.002	0.085
RS Calcium oxalate	3.183 ± 1.719	2.474 ± 1.514	0.009	3.668 ± 1.995	2.652 ± 1.663	0.003	0.210

Abbreviations: C, control; MR, meal replacement; RS, relative supersaturation; SD, standard deviation. ^a^ *p*-value: Wilcoxon-test within groups. ^b^ *p*-value: Mann–Whitney-U-test.

## Data Availability

The data presented in this study are available upon reasonable personal request.
